# Supplementary material for: Use of hybrid quantum-classical algorithms for enhancing biomarker classification
Source: PLoS One. 2025 Jul 17;20(7):e0327928. doi: 10.1371/journal.pone.0327928 (PMC12270134; doi:10.1371/journal.pone.0327928)
Supplement: S3 File — (DOCX) [file pone.0327928.s003.docx]

Supplementary file 3 – The results of the 10 performance metrics of the miRNA biomarker study (NT vs M0) for the four case studies are as follows: SVC, QSVC, NQE+SVC, and NQE+QSVC.

| **Metrics** | **SVM** | **QSVM** | **NQE+SVC** | **NQE+QSVC** |
| --- | --- | --- | --- | --- |
| Sensitivity/Recall, TPR | 0.73 | **0.92** | 0.91 | **0.93** |
| Specificity, SPC | **0.77** | 0.73 | 0.68 | **0.73** |
| Precision, PPV | 0.89 | **0.9** | 0.88 | **0.90** |
| F1 Score | 0.80 | **0.91** | 0.90 | **0.92** |
| Accuracy | 0.74 | **0.86** | 0.85 | **0.87** |
| Negative Predictive Value | 0.51 | **0.76** | 0.75 | **0.8** |
| False Positive Rate* | 0.23 | **0.27** | 0.32 | **0.27** |
| False Discovery Rate* | **0.10** | **0.10** | 0.11 | **0.09** |
| False Negative Rate* | 0.27 | **0.08** | **0.08** | 0.07 |
| Matthews Correlation Coefficient | 0.45 | **0.65** | 0.62 | **0.68** |
| Comparative score | 1+1-tier | **8**+1-tier | 1 | **9** |

* A lower value implies better performance
