# Supplementary material for: Use of hybrid quantum-classical algorithms for enhancing biomarker classification
Source: PLoS One. 2025 Jul 17;20(7):e0327928. doi: 10.1371/journal.pone.0327928 (PMC12270134; doi:10.1371/journal.pone.0327928)
Supplement: S4 File — (DOCX) [file pone.0327928.s004.docx]

Supplementary file 4 – The results of the 10 performance metrics of the miRNA biomarker study (M0 vs M1) for the two case studies are as follows: NQE+SVC and NQE+QSVC.

|  | **4 PCs** | | **5 PCs** | | **6 PCs** | |
| --- | --- | --- | --- | --- | --- | --- |
| Metrics | NQE+SVC | NQE+QSVC | NQE+SVC | NQE+QSVC | NQE+SVC | NQE+QSVC |
| Sensitivity/Recall, TPR | 0.689$\pm0.13,$  0.615[test] | 0.674$\pm0.1661,$  0.46[test] | 0.635$\pm0.054,$  0.385[test] | 0.53$\pm0.1,$  0.385[test] | 0.745$\pm0.134,$  0.231 [test] | 0.694$\pm0.147,$  0.615 [test] |
| Specificity, SPC | 0.527$\pm0.109,$  0.733 | 0.487  $\pm0.16$,  0.60 | 0.65$\pm0.098,$  0.556 | 0.679$\pm0.136,$  0.644 | 0.701$\pm0.054,$  0.889 | 0.630$\pm0.14,$  0.733 |
| Precision, PPV | 0.59$\pm0.026,$  0.400 | 0.574$\pm0.0713,$  0.25 | 0.649$\pm0.082,$  0.200 | 0.634$\pm0.098,$  0.238 | 0.712$\pm0.072,$  0.375 | 0.657$\pm0.084,$  0.400 |
| F1 Score | 0.629$\pm0.063,$  0.485 | 0.607$\pm0.089,$  0.324 | 0.635$\pm0.049,$  0.263 | 0.57$\pm0.078,$  0.294 | 0.724$\pm0.097,$  0.286 | 0.668$\pm0.095,$  0.485 |
| Accuracy | 0.608$\pm0.029,$  0.707 | 0.58$0\pm0.063,$  0.569 | 0.64$\pm0.054,$  0.517 | 0.604$\pm0.074,$  0.586 | 0.724$\pm0.078,$  0.741 | 0.66$\pm0.088,$  0.707 |
| Negative Predictive Value (NPV) | 0.645$\pm0.042,$  0.868 | 0.613$\pm0.093,$  0.974 | 0.653$\pm0.043,$  0.758 | 0.59$\pm0.069,$  0.784 | 0.75$\pm0.103,$  0.80 | 0.685$\pm0.12,$  0.868 |
| False Positive Rate (FPR)* | 0.470$\pm0.109,$  0.267 | 0.513  $\pm0.165$  0.400 | 0.349$\pm0.098,$  0.444 | 0.32$\pm0.136,$  0.356 | 0.298$\pm0.054,$  0.111 | 0.369$\pm0.139,$  0.267 |
| False Discovery Rate (FDR)* | 0.409$\pm0.026,$  0.600 | 0.425$\pm0.071,$  0.750 | 0.350$\pm0.082,$  0.800 | 0.36$\pm0.098,$  0.762 | 0.287$\pm0.072,$  0.625 | 0.343$\pm0.084,$  0.600 |
| False Negative Rate (FNR)* | 0.311$\pm0.13,$  0.385 | 0.330$\pm0.166,$  0.538 | 0.365$\pm0.054,$  0.615 | 0.47$\pm0.104,$  0.615 | 0.254$\pm0.13,$  0.769 | 0.306$\pm0.147,$  0.385 |
| Matthews Correlation Coefficient | 0.225$\pm0.061,$  0.306 | 0.173$\pm0.134,$  0.052 | 0.294$\pm0.114,$  -0.05 | 0.216$\pm0.15,$  0.025 | 0.455$\pm0.163,$  0.145 | 0.330$\pm0.18,$  0.306 |
| **Comparative Points** | **10** | **0** | **0** | **10** | **3** | **7** |
