# Supplementary material for: Use of hybrid quantum-classical algorithms for enhancing biomarker classification
Source: PLoS One. 2025 Jul 17;20(7):e0327928. doi: 10.1371/journal.pone.0327928 (PMC12270134; doi:10.1371/journal.pone.0327928)
Supplement: S5 File — (DOCX) [file pone.0327928.s005.docx]

Supplementary file 5 – The results of the 10 performance metrics of the miRNA biomarker study (M0 vs M1) for the two case studies are as follows: NQE+NN, and NQE+QNN.

|  | 4 PCs | | 5 PCs | | 6 PCs | |
| --- | --- | --- | --- | --- | --- | --- |
| Metrics | NN | QNN | NN | QNN | NN | QNN |
| Sensitivity/Recall, TPR | 0.8158  ±0.0914  0.3846 | 0.6991  ±0.1308  0.6923 | 0.6754  ±0.2064  0.6154 | 0.5798  ±0.1173  0.3846 | 0.8246  ±0.1523  0.2308 | 0.6254  ±0.1428  0.5385 |
| Specificity, SPC=TNR | 0.6193  ±0.1461  0.7111 | 0.5956  ±0.1068  0.4444 | 0.5895  ±0.2187  0.5111 | 0.5693  ±0.1546  0.6222 | 0.7719  ±0.1091  0.8222 | 0.5325  ±0.135  0.6222 |
| Precision, PPV | 0.6919  ±0.0745  0.2778 | 0.6346  ±0.0743  0.2647 | 0.6421  ±0.1162  0.2667 | 0.581  ±0.1061  0.2273 | 0.7858  ±0.1017  0.2727 | 0.5749  ±0.0739  0.2917 |
| F1 Score | 0.7429  ±0.0531  0.3226 | 0.6604  ±0.0912  0.383 | 0.6367  ±0.1126  0.3721 | 0.5754  ±0.0976  0.2857 | 0.7993  ±0.1093  0.25 | 0.5927  ±0.0878  0.3784 |
| Accuracy | 0.7175  ±0.0644  0.6379 | 0.6474  ±0.0771  0.5 | 0.6325  ±0.0969  0.5345 | 0.5746  ±0.0992  0.569 | 0.7983  ±0.1  0.6897 | 0.5789  ±0.0798  0.6034 |
| Negative Predictive Value | 0.7764  ±0.0728  0.8 | 0.6732  ±0.0921  0.8333 | 0.6713  ±0.155  0.8214 | 0.5722  ±0.1063  0.7778 | 0.8321  ±0.1326  0.7872 | 0.5978  ±0.1185  0.8235 |
| False Positive Rate (FPR)* | 0.3807  ±0.1461  0.2889 | 0.4044  ±0.1068  0.5556 | 0.4105  ±0.2187  0.4889 | 0.4307  ±0.1546  0.3778 | 0.2281  ±0.1091  0.1778 | 0.4675  ±0.135  0.3778 |
| False Discovery Rate (FDR)* | 0.3082  ±0.0745  0.7222 | 0.3654  ±0.0743  0.7353 | 0.3579  ±0.1162  0.7333 | 0.419  ±0.1061  0.7727 | 0.2142  ±0.1017  0.7273 | 0.4251  ±0.0739  0.7083 |
| False Negative Rate (FNR)* | 0.1842  ±0.0914  0.6154 | 0.3009  ±0.1308  0.3077 | 0.3246  ±0.2064  0.3846 | 0.4202  ±0.1173  0.6154 | 0.1754  ±0.1523  0.7692 | 0.3746  ±0.1428  0.4615 |
| Matthews Correlation Coefficient | 0.451  ±0.1227  0.0863 | 0.3011  ±0.1556  0.1158 | 0.2865  ±0.2053  0.1056 | 0.1511  ±0.2025  0.0059 | 0.6069  ±0.2026  0.0564 | 0.1647  ±0.1682  0.1361 |
| Comparative scores | 5 | 5 | 7 | 3 | 3 | **7** |
