# Supplementary material for: Use of hybrid quantum-classical algorithms for enhancing biomarker classification
Source: PLoS One. 2025 Jul 17;20(7):e0327928. doi: 10.1371/journal.pone.0327928 (PMC12270134; doi:10.1371/journal.pone.0327928)
Supplement: S1 Fig — (DOCX) [file pone.0327928.s006.docx]

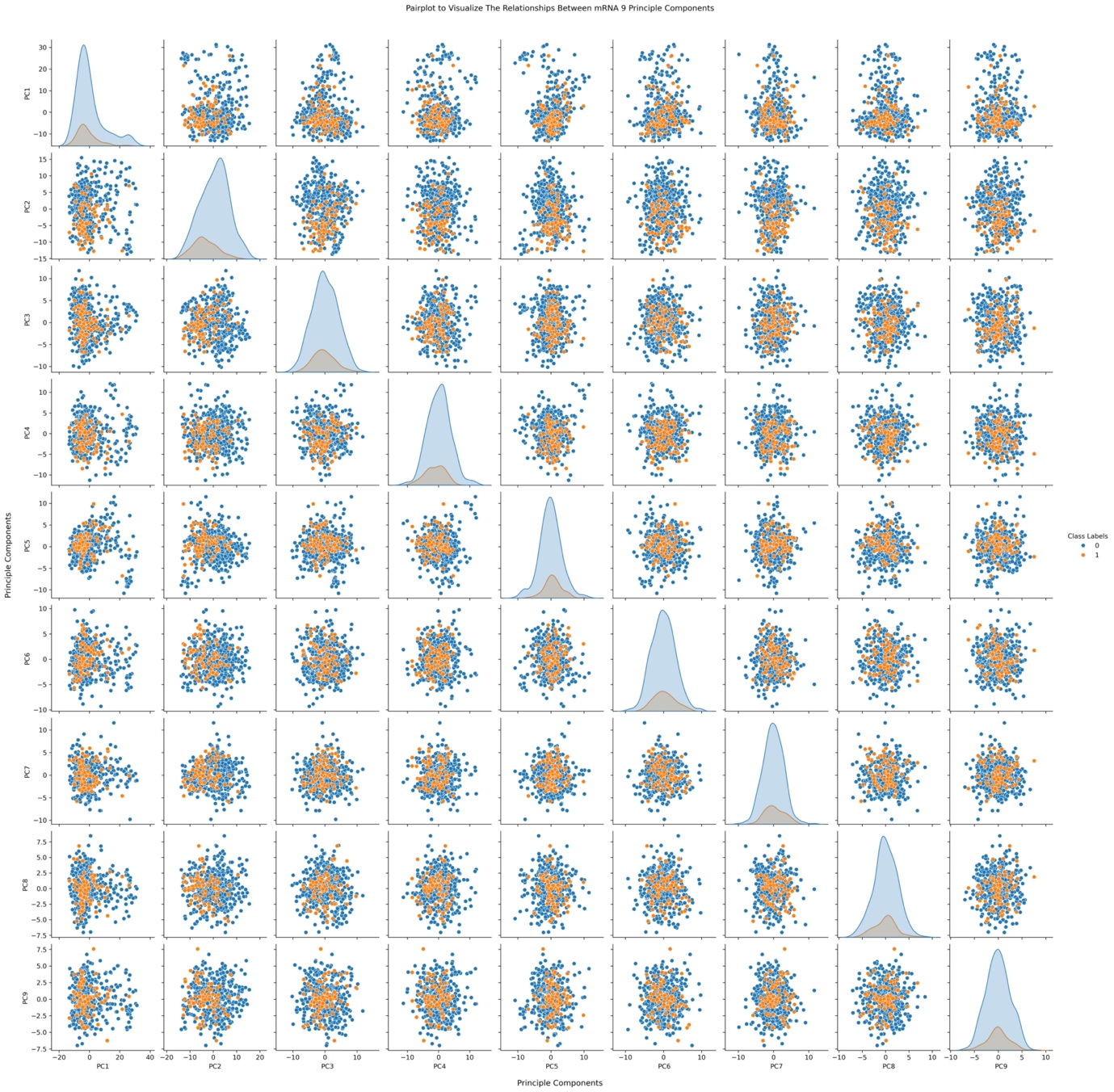


The PCA plots of the data distribution (mRNA) projected onto two principal components selected from PC1 through PC9.
