# Supplementary figures and images for: Use of hybrid quantum-classical algorithms for enhancing biomarker classification
Source: PLoS One. 2025 Jul 17;20(7):e0327928. doi: 10.1371/journal.pone.0327928 (PMC12270134; doi:10.1371/journal.pone.0327928)

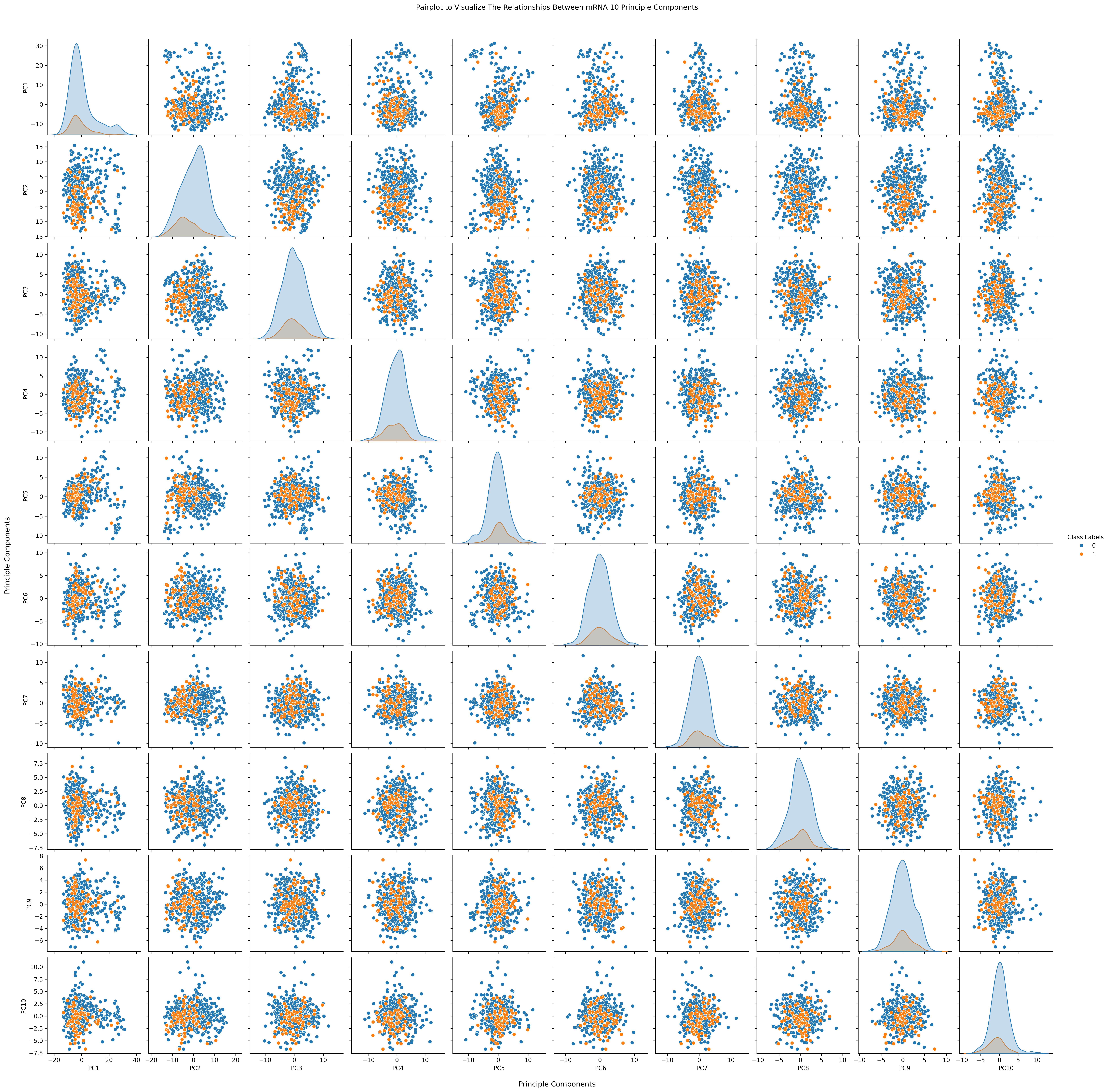

Supplement: S2 Fig — (JPEG) [file pone.0327928.s007.jpeg]

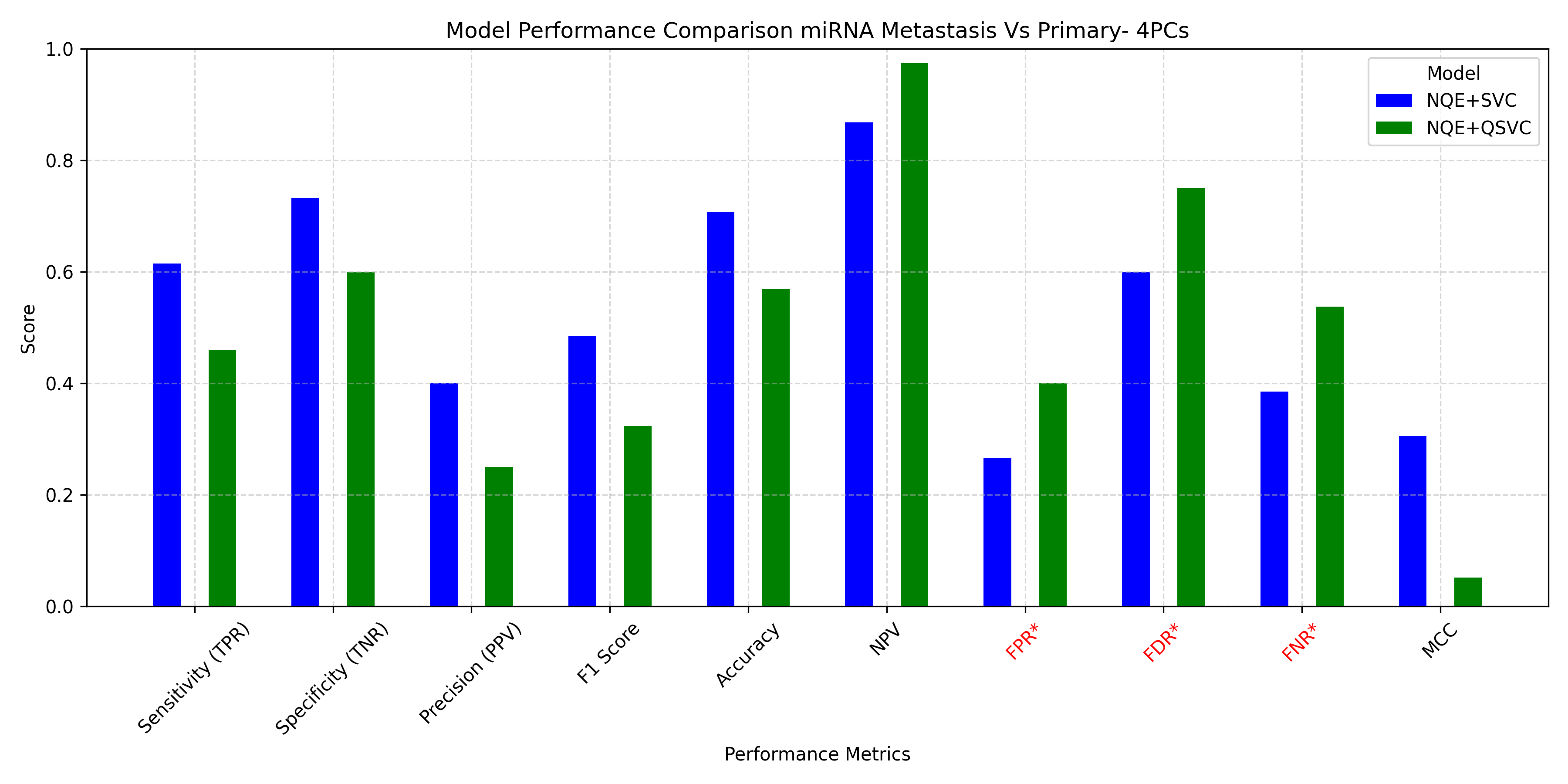

Supplement: S5 Fig — (ZIP) [file pone.0327928.s010.zip › Supplementary figure 5 the 10 metrics for miRNA TPvsM1 4PCs.png]

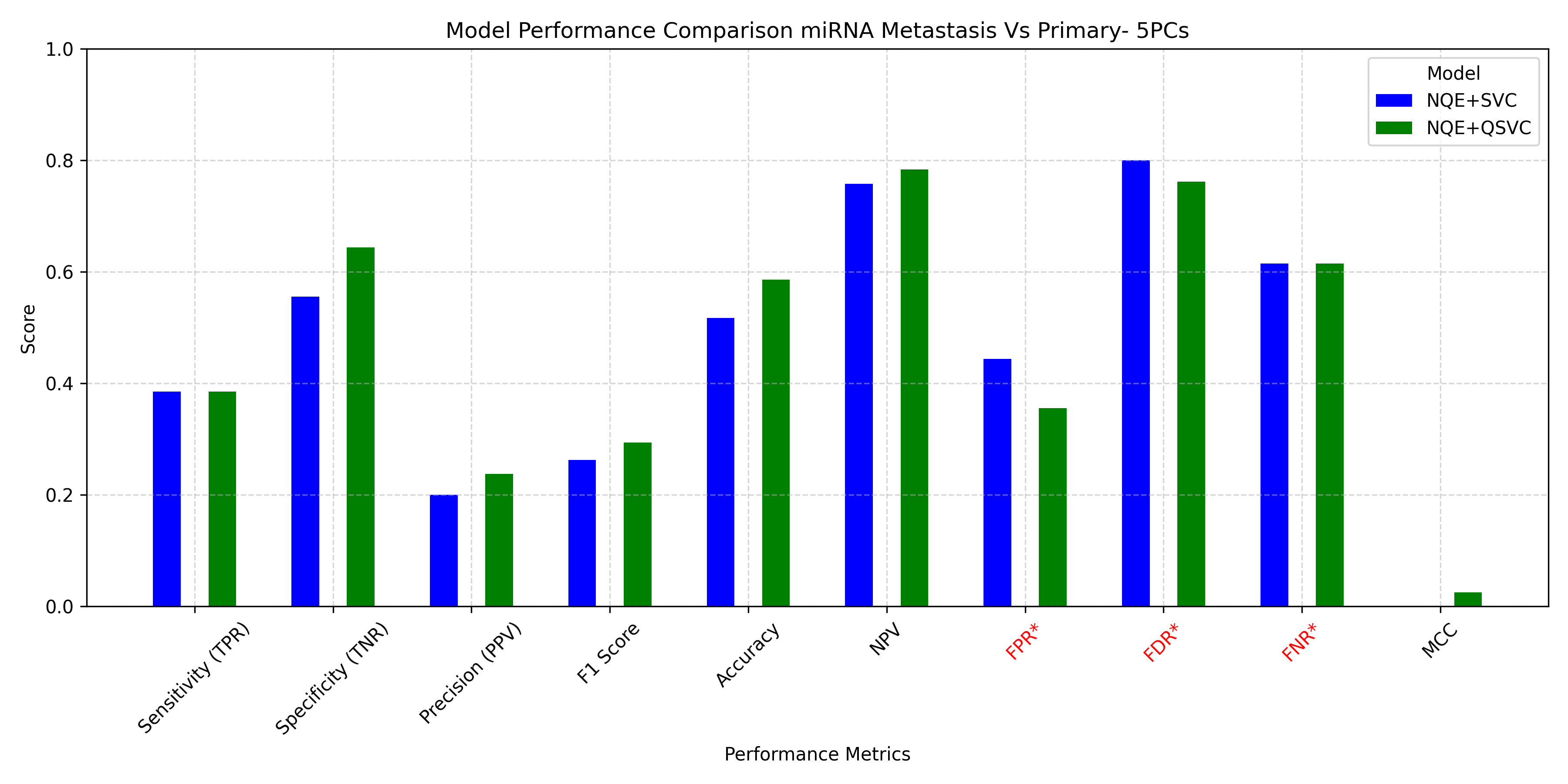

Supplement: S6 Fig — (PNG) [file pone.0327928.s011.png]

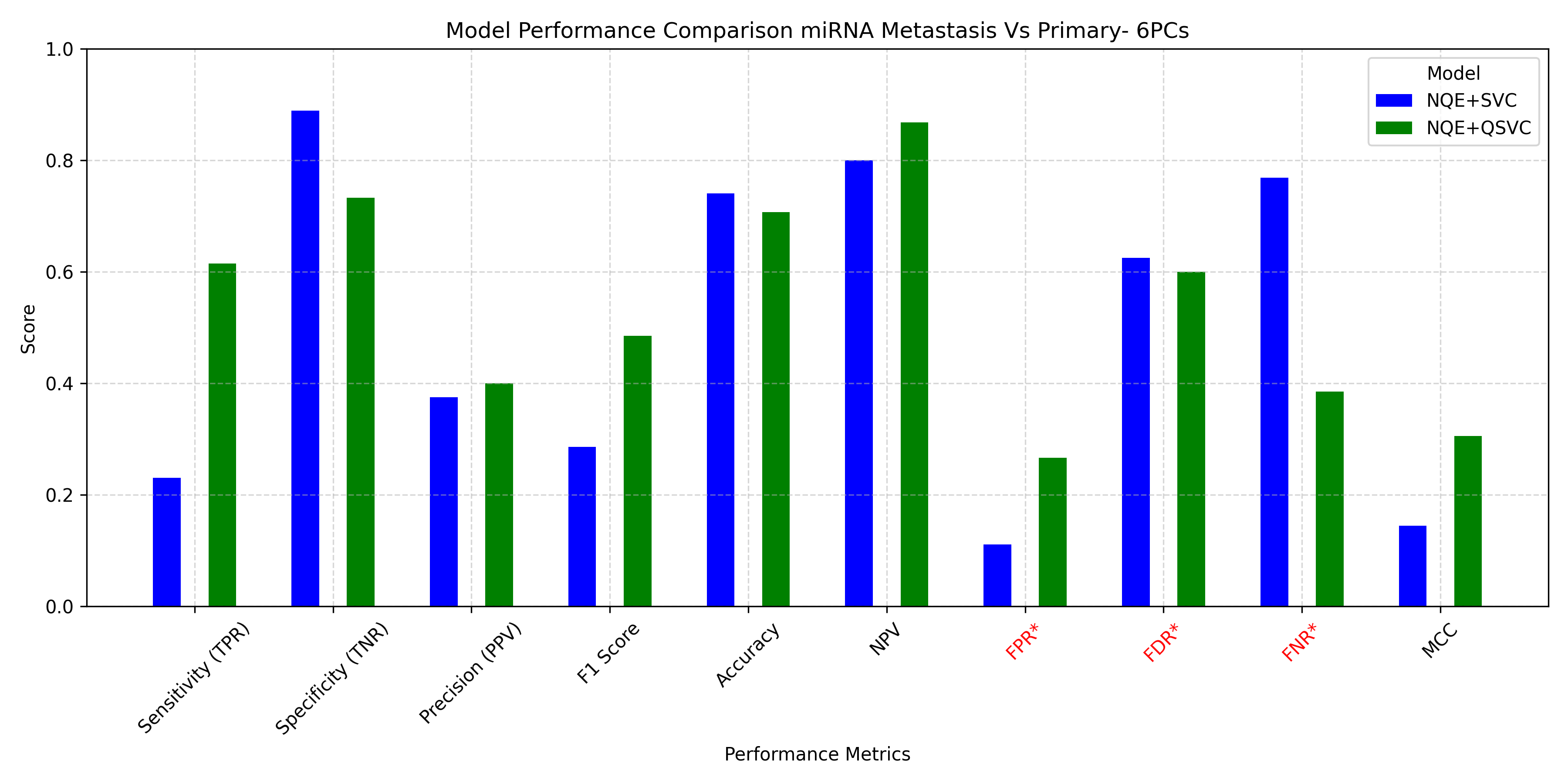

Supplement: S7 Fig — (PNG) [file pone.0327928.s012.png]

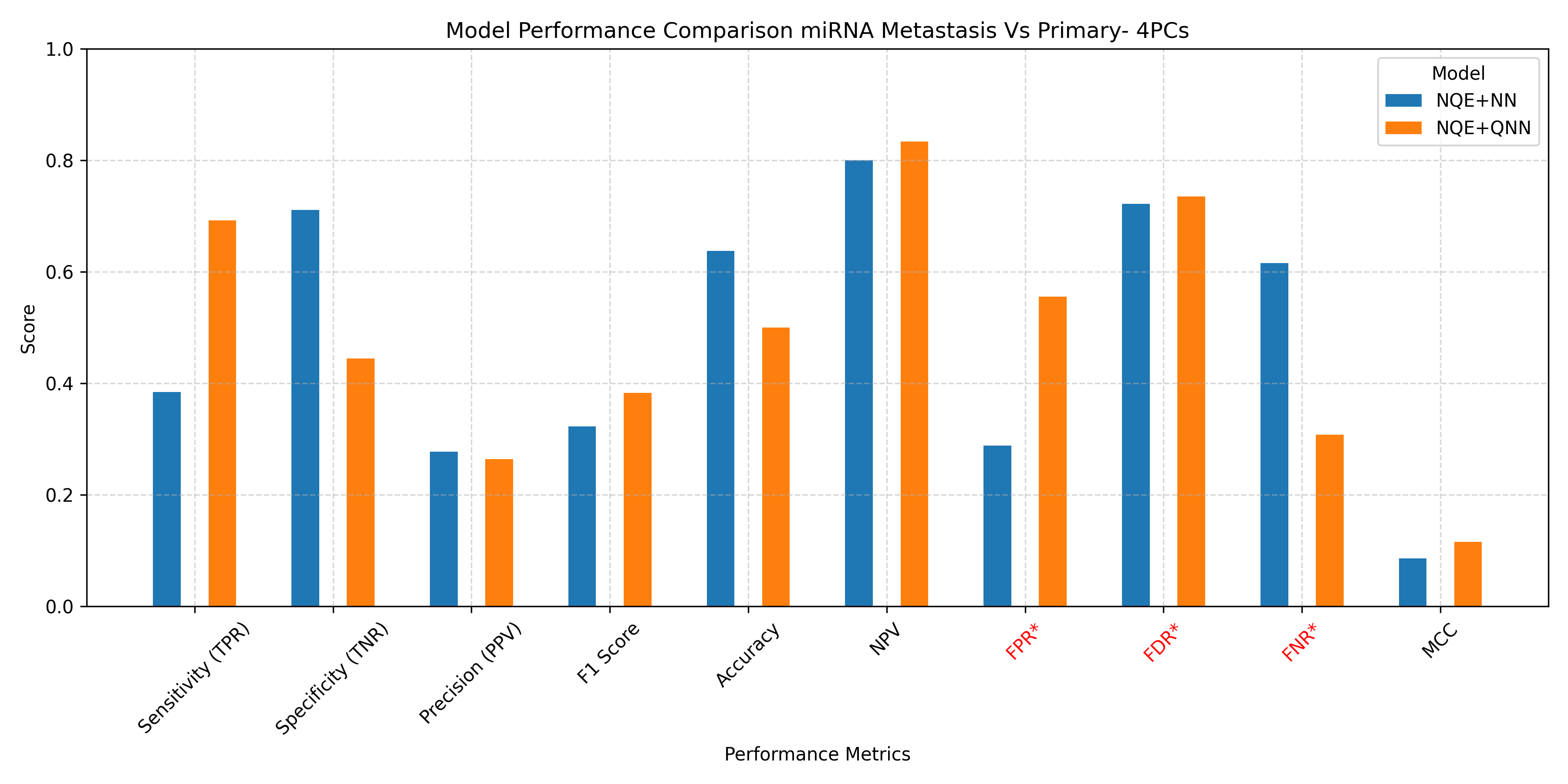

Supplement: S8 Fig — (PNG) [file pone.0327928.s013.png]

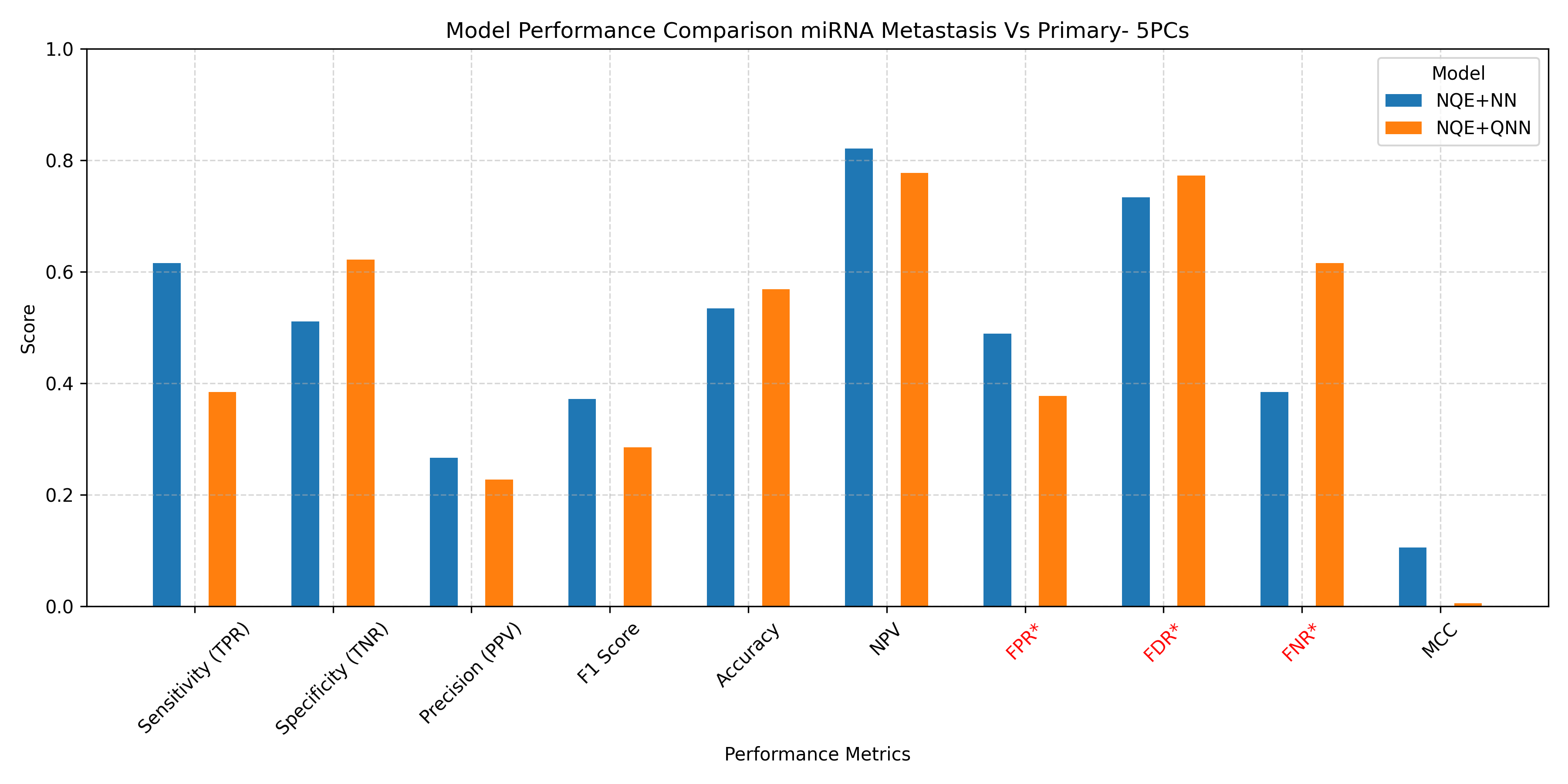

Supplement: S9 Fig — (PNG) [file pone.0327928.s014.png]

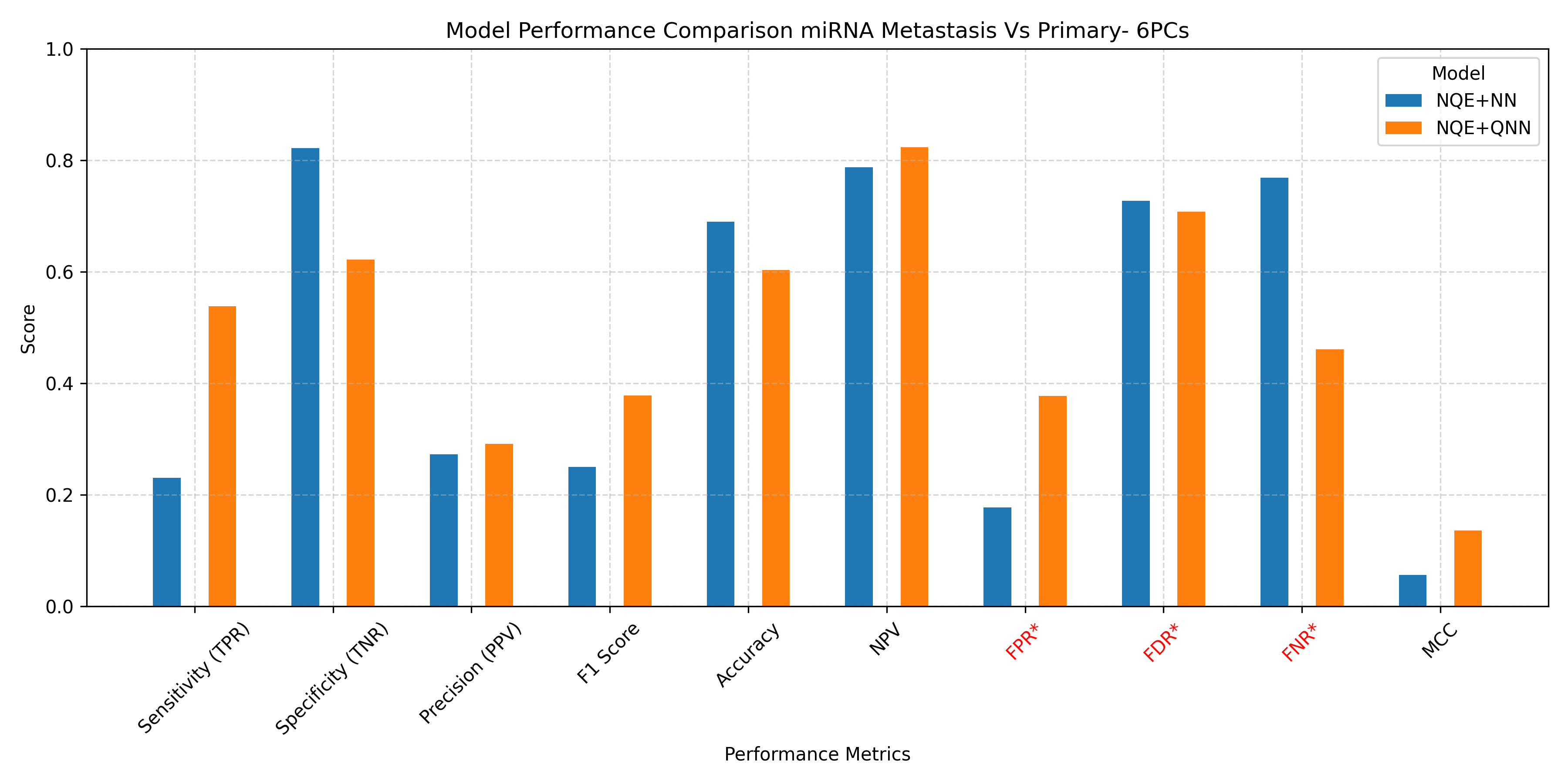

Supplement: S10 Fig — (PNG) [file pone.0327928.s015.png]
