# Supplementary material for: Use of hybrid quantum-classical algorithms for enhancing biomarker classification
Source: PLoS One. 2025 Jul 17;20(7):e0327928. doi: 10.1371/journal.pone.0327928 (PMC12270134; doi:10.1371/journal.pone.0327928)
Supplement: S5 Fig — (ZIP) [file pone.0327928.s010.zip › Supplementary figure 5 pairplot miRNA.docx]

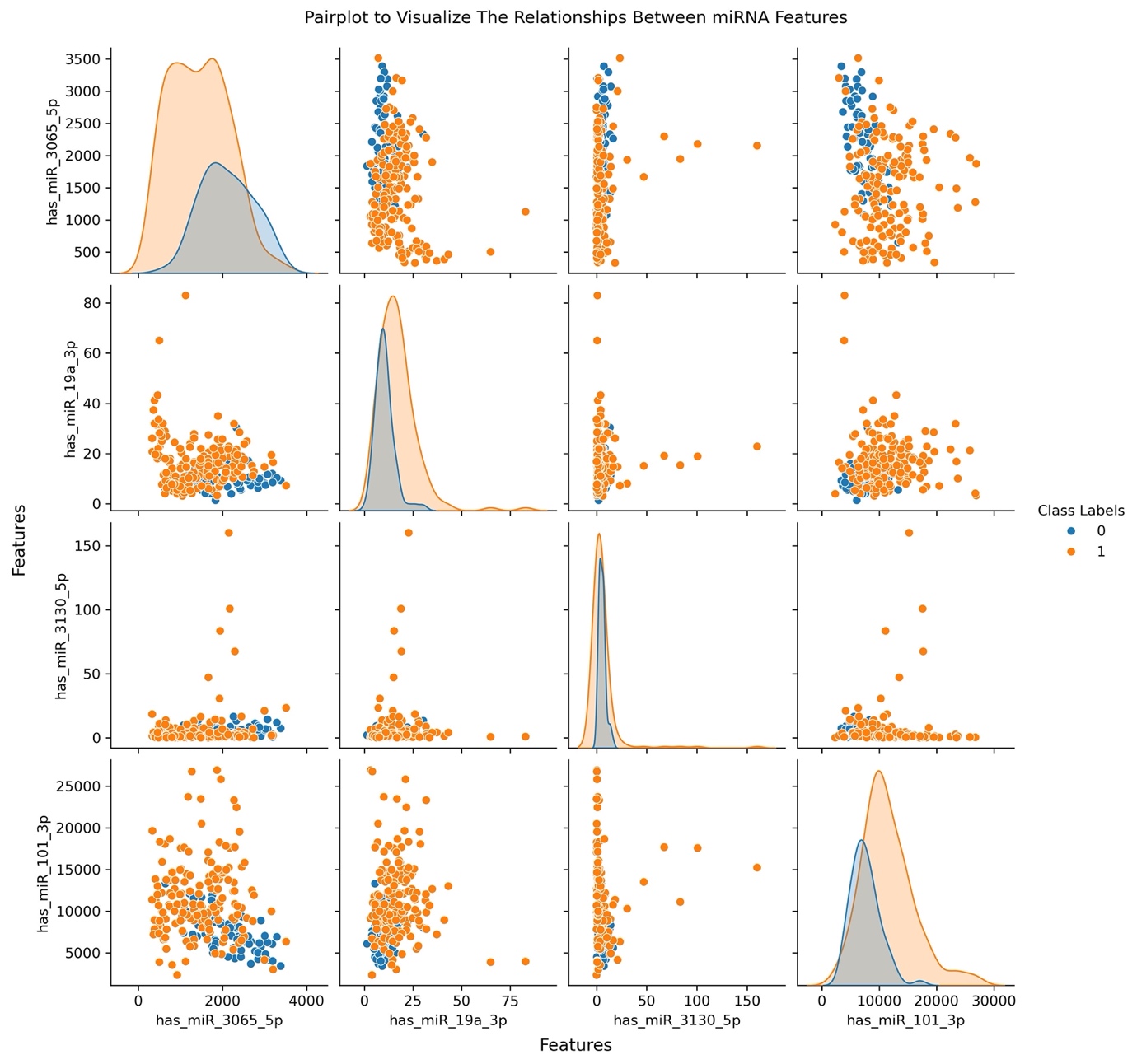


The plots of the data distribution (miRNA) projected onto two selected features out of four possible features.
