# Supplementary material for: Use of hybrid quantum-classical algorithms for enhancing biomarker classification
Source: PLoS One. 2025 Jul 17;20(7):e0327928. doi: 10.1371/journal.pone.0327928 (PMC12270134; doi:10.1371/journal.pone.0327928)
Supplement: Attachment — Submitted filename: Response to Reviewers-v2.docx [file pone.0327928.s017.docx]

Reviewers' comments:
Reviewer's Responses to Questions

**Comments to the Author**
1. Is the manuscript technically sound, and do the data support the conclusions?
The manuscript must describe a technically sound piece of scientific research with data that supports the conclusions. Experiments must have been conducted rigorously, with appropriate controls, replication, and sample sizes. The conclusions must be drawn appropriately based on the data presented.

Reviewer #1: Partly

Reviewer #2: Partly the

**Reply**

**We thank the reviewers for their helpful suggestions, which have contributed to improving our work.**

**The manuscript presents a technically sound piece of work addressing several key challenges. Specifically, it: (i) tackles the data imbalance issue in a biomedical dataset, where (ii) integrates a data embedding method, Neural Quantum Embedding (NQE), to improve the performance of a quantum classifier (QSVC), benchmarking it for NQE+SVC with 4 to 6 principal components (PCs) and 9 to 12 PCs, (iii) optimizes all models through the exploration of various hyperparameter combinations, ensuring a fair and unbiased comparison across them, and (iv) employs 10 performance metrics to evaluate classification accuracy, mitigating potential overfitting issues.**

**In the revised version of the manuscript, we extend the above analysis by incorporating NQE with the quantum version of the neural network (QNN) classifier and comparing its performance with NQE and a traditional NN classifier. Again, we have demonstrated after introducing NQE it can significantly enhances the classification performance.**

**In the revised manuscript, we expand upon the previous analysis by incorporating NQE with the quantum version of the neural network (QNN) classifier and comparing its performance to the NQE-enhanced NN classifiers. Once again, our findings demonstrate that the introduction of NQE significantly enhances classification performance.**

**In summary, our approach demonstrates that using NQE significantly enhances the performance of quantum classifiers in biomedical research.**

2. Has the statistical analysis been performed appropriately and rigorously?

Reviewer #1: No

Reviewer #2: Yes

**Reply**

**In the revised version of the manuscript, we present the results of 10 performance metrics, along with their standard deviations, for the training set across four cases: NQE-enhanced NN, QNN, SVC, and QSVC, each using 4 to 6 PCs. This allows us to assess the robustness of the results for the 10 performance metrics.**

3. Have the authors made all data underlying the findings in their manuscript fully available?
The [PLOS Data policy](http://track.editorialmanager.com/CL0/http:%2F%2Fwww.plosone.org%2Fstatic%2Fpolicies.action%23sharing/1/010f0192fe46903d-c7004a41-df1f-4cc0-997b-cb76a377d91f-000000/3ZqAocgOFjZlfp5-d8ctB4gqjWbvCQKY7EO4hK3j-Tc=183) requires authors to make all data underlying the findings described in their manuscript fully available without restriction, with rare exception (please refer to the Data Availability Statement in the manuscript PDF file). The data should be provided as part of the manuscript or its supporting information, or deposited to a public repository. For example, in addition to summary statistics, the data points behind means, medians and variance measures should be available. If there are restrictions on publicly sharing data—e.g. participant privacy or use of data from a third party—those must be specified.

Reviewer #1: Yes

Reviewer #2: Yes

**Reply**

**Thank you for acknowledging our contributions.**

4. Is the manuscript presented in an intelligible fashion and written in standard English?
PLOS ONE does not copyedit accepted manuscripts, so the language in submitted articles must be clear, correct, and unambiguous. Any typographical or grammatical errors should be corrected at revision, so please note any specific errors here.

Reviewer #1: Yes

Reviewer #2: No.

**Reply**

**In the revised manuscript, the 'Introduction' section has been expanded to provide more detailed explanations of the underlying principles of quantum computing, aiming to make the content accessible to a broader readership. Additionally, the 'Materials and Methods' and ‘Results’ sections have been updated, with a more detailed explanation of Figures 1 to 4.**

5. Review Comments to the Author
Please use the space provided to explain your answers to the questions above. You may also include additional comments for the author, including concerns about dual publication, research ethics, or publication ethics. (Please upload your review as an attachment if it exceeds 20,000 characters)

Reviewer #1: This paper presents an innovative application of quantum machine learning (QML) in cancer biomarker classification. The paper explores the use of a hybrid quantum-classical approach to improve biomarker classification in metastatic cancer, specifically clear cell renal cell carcinoma (ccRCC). The authors aim to enhance the performance (accuracy and execution time) of the Quantum Support Vector Classifier (QSVC) by integrating it with Neural Quantum Embedding (NQE) and compare its performance with a classical Support Vector Classifier (SVC).
This paper focuses on evaluating the effectiveness of QSVC, combined with NQE, for binary classification of cancer data.

The integration of NQE and QSVC is an innovative approach, particularly for biomedical datasets like gene expression and miRNA profiles related to cancer metastasis. This combination of quantum and classical techniques shows promise for complex classification tasks but broader dataset testing and better explanations of quantum principles could enhance its impact.

While the paper presents a novel and promising approach, but in order to strengthen the paper and address potential concerns from readers, I recommend a major revision aim to clarify certain aspects of the methodology, improve transparency, and provide a deeper understanding of presented concepts:

1. Expand on Quantum Concepts: While the research is highly technical and relevant to a specialized audience, it may be too complex for a broader readership. There could be more effort in explaining the underlying quantum principles for readers less familiar with quantum computing, which would make the paper more accessible.

**Reply
In the revised manuscript we have included more detailed descriptions to explain the underlying principles in quantum computing.**

**Quantum machine learning (QML) [8] is an emerging and popular research field that integrates the concepts of quantum computing and machine learning. This integration leverages the unique capabilities of quantum computers to enhance and accelerate machine learning tasks. The most commonly used model in quantum computing is the gate-based quantum computation model [9]. Essentially the model can be described in three steps: (i) Initialization: Qubits are initialized in a specific quantum state, often the |0> state. (ii) Encoding: Input data is encoded into the quantum amplitudes of these qubits. (iii) Quantum Gates: Quantum unitary gates are applied to transform the quantum states of the qubits. These gates can operate on multiple qubits simultaneously, a property known as quantum parallelism.**

**By designing and implementing quantum circuits (quantum algorithms) appropriately, the quantum states undergo interference. This interference enhances the amplitudes of the desired solutions. The final solution is obtained by performing measurements on the quantum states. Due to the inherent randomness in quantum measurements, multiple repetitions are necessary to determine the required solution with high probability.**

**Quantum computing excels in solving specific types of problems, such as quantum simulations in physics and chemistry. However, it is not expected to replace classical computing entirely. Instead, a hybrid approach that combines classical and quantum computing is often used to tackle complex problems more efficiently [10-12].**

2. Could you provide more detailed explanations or visual aids to clarify how the quantum embedding circuit (Figure 3) functions for readers unfamiliar with quantum mechanics? A simplified diagram or intuitive description might enhance accessibility.

**Reply**

**Figure 3 illustrates the quantum embedding circuit. Its objective is to train the input features in a way that maximizes the separability between the M0 and M1 class labels. The number of qubits corresponds to the number of input features (or PCs). The embedding process employs Hadamard (H) gates, rotation (RZ) gates, and control-NOT (CNOT) gates, applied three times. These gates serve the following purposes: (i) creating superposition in the input quantum qubits, (ii) parametrizing the states by applying phase shifts, which are essential for generating interference among the quantum states, and (iii) introducing entanglement between the qubits.**

**To reverse the embedding, a layer of the inverse of the RZ gates is applied in reverse order. The two mapping functions, 𝜑𝑘 and 𝜑𝑗𝑘 (as shown in the function 𝑉(𝑔(𝒙, 𝒘) ) in Figure 2) are defined to be 2xk and (π - xj)(π - xk) respectively.**

**We have also revised the figure caption as follows:**

**Figure 3. The quantum embedding circuit designed for the miRNA dataset, featuring three layers of repeated Hadamard (H), RZ, and control-NOT unitary gates. To conserve space, a partial depiction of the inverse RZ gates applied in reverse order is shown.**

^^^^^^^^^^^^^^^^^^^^^^^^^^^^^^^^^^^^^^^^^^^^^^^^^^^^^^^^^^^^^^^^^^^^^^^^^^^

3. Could you explain more intuitively how the quantum embedding circuit contributes to the improvement in classification performance? For readers unfamiliar with quantum computing, an intuitive description would be helpful.

**Reply**

We have provided a more detailed description of the quantum embedding circuit in the revised manuscript. This additional information has been included after the following paragraph: ‘The Neural Quantum Embedding (NQE) approach … capable of separating the  classes of data into orthogonal subspaces.’

**In other words, the input features are non-linearly mapped to a higher-dimensional Hilbert space using a feature mapping function, which consists of an exponential operator made up of non-linear functions and the Pauli Z operators. The initial two input samples are encoded into quantum states, and the resulting states are measured to determine whether the two samples share the same class label. This overlap is quantified by the fidelity function. The fidelity result is then used to update the neural network's weighting factors, optimizing the separability of the two classes of data into orthogonal subspaces, thereby maximizing their difference.**

^^^^^^^^^^^^^^^^^^^^^^^^^^^^^^^^^^^^^^^^^^^^^^^^^^^^^^^^^^^^^^^^^^^^^^^^^^^

4. Why did you specifically choose the QSVC and NQE hybrid approach for your study? While the study compares QSVC and SVC, it would benefit from comparing additional classical machine learning methods (e.g., random forest, neural networks) to better position the advantages of the hybrid approach.

**Reply**

The title of the article has been changed to "Use of Hybrid Quantum-Classical Algorithms for Enhancing Biomarker Classification" to reflect the inclusion of the quantum neural network algorithm in our study. Below are the updated contents of the article.

**^^^^^^^^^^^^^^^^^^^^^^^^^^^^^^^^^^^^^^^^^^^^^^^^^^^^^^^**

**In addition to QSVC, quantum versions of several machine learning algorithms have been developed, including QNN (quantum neural network) [39] and QCNN (quantum convolutional neural network) [40]. Both QSVC and QNN classifiers are considered in the work.**

**The use of QCNN combined with NQE for classifying the digits 0 and 1 within the MNIST dataset has been reported. It was found that the classification accuracy of QCNN trained with NQE significantly improves compared to QCNN without NQE training. However, there are no studies on the use of NQE+QSVC and NQE+QNN for classification problems. We explore this possibility using biomedical data. We noted that the quantum version of random forest (QRF) is currently not available.**

**For both QSVC and QNN, the key factor that determines the success of classifcation performance is the choice of an appropriate feature map. If the feature map is not well-suited to the data, it will hinder the performance of the entire machine learning model [42].**

**To evaluate the impact of NQE on classfication performance other than SVC, we have included studies on NQE+NN and NQE+QNN in this work, utilizing 5-fold cross-validation. The workflow diagram of this study is similar to that in Figure 1, with SVC/QSVC replaced by NN/QNN; therefore, the diagram is omitted.** **For the NN classiifier, the following three hyperparameters were optimized: the number of hiddden layers, learning rate, and epochs. For the QNN classiifier (SampleQNN), the following three hyperparameters were optimized: the number epochs, the number of ZZfeatureMap repetitions, and the number of ansatz layers. The NQE+NN and NQE+QNN models were then applied to the test set.**

**Table 3 presents the results of the 10 performance metrics for the training set, including standard deviation, after selecting the optimal cross-entropy value via 5-fold cross-validation. The final row of Table 3 provides the comparative scores across the 10 metrics using the ‘test’ set results, where higher values correspond to better performance. We observed that NQE+NN outperforms NQE+QNN when using 4 and 5 PCs. However, NQE+QNN demonstrates superior performance with 6 PCs, as indicated by the comparative score of 0 versus 10. These findings suggest that NQE has the potential to enhance the performance of the QNN classifiers. Furthermore, all standard deviation values are relatively small compared to the mean values, except in two instances (NQE+NN, 6 PCs, FPR, and FNR), indicating that the results for the 10 performance metrics are robust.**

**We also found a significant difference between the performance metric values for the training and test sets, particularly for PPV, F1, FDR, FNR, and MCC. As suggested earlier, the issue of data imbalance remains an intrinsically challenging problem.**

Table 3. Classification performance results using 5-fold cross-validation for the training datasets after applying the data imbalance tool, SMOTE. The values without the '±' signs represent the results for the ‘test’ set.

|  | 4 PCs | | 5 PCs | | 6 PCs | |
| --- | --- | --- | --- | --- | --- | --- |
| Metrics | NQE+NN | NQE+QNN | NQE+NN | NQE+QNN | NQE+NN | NQE+QNN |
| Sensitivity/Recall, TPR | 0.727  ±0.064  0.433 | 0.676  ±0.056  0.433 | 0.812  ±0.045  0.567 | 0.73  ±0.08  0.467 | 0.772  ±0.104  0.533 | 0.641  ±0.09  0.633 |
| Specificity, SPC=TNR | 0.799  ±0.078  0.725 | 0.766  ±0.07  0.681 | 0.733  ±0.086  0.623 | 0.706  ±0.082  0.667 | 0.687  ±0.103  0.449 | 0.632  ±0.077  0.645 |
| Precision, PPV | 0.79  ±0.057  0.255 | 0.748  ±0.052  0.228 | 0.758  ±0.055  0.246 | 0.717  ±0.047  0.233 | 0.717  ±0.051  0.174 | 0.637  ±0.042  0.279 |
| F1 Score | 0.754  ±0.033  0.321 | 0.708  ±0.035  0.299 | 0.782  ±0.029  0.343 | 0.72  ±0.046  0.311 | 0.738  ±0.054  0.262 | 0.636  ±0.053  0.388 |
| Accuracy | 0.763  ±0.031  0.673 | 0.721  ±0.033  0.637 | 0.772  ±0.036  0.613 | 0.718  ±0.039  0.631 | 0.73  ±0.047  0.464 | 0.637  ±0.04  0.643 |
| Negative Predictive Value | 0.748  ±0.035  0.855 | 0.704  ±0.033  0.847 | 0.797  ±0.031  0.869 | 0.727  ±0.048  0.852 | 0.762  ±0.074  0.816 | 0.642  ±0.05  0.89 |
| False Positive Rate (FPR)* | 0.201  ±0.078  0.275 | 0.234  ±0.07  0.319 | 0.267  ±0.086  0.377 | 0.294  ±0.082  0.333 | 0.313  ±0.103  0.551 | 0.368  ±0.077  0.355 |
| False Discovery Rate (FDR)* | 0.21  ±0.057  0.745 | 0.252  ±0.052  0.772 | 0.242  ±0.055  0.754 | 0.283  ±0.047  0.767 | 0.283  ±0.051  0.826 | 0.363  ±0.042  0.721 |
| False Negative Rate (FNR)* | 0.273  ±0.064  0.567 | 0.324  ±0.056  0.567 | 0.188  ±0.045  0.433 | 0.27  ±0.08  0.533 | 0.228  ±0.104  0.467 | 0.359  ±0.09  0.367 |
| Matthews Correlation Coefficient | 0.532  ±0.062  0.132 | 0.447  ±0.068  0.093 | 0.549  ±0.068  0.148 | 0.44  ±0.078  0.107 | 0.468  ±0.092  -0.013 | 0.276  ±0.082  0.217 |
| Comparative scores | 8+2-tier | 2- tier | 7 | 3 | 0 | 10 |

* A lower value implies better performance

^^^^^^^^^^^^^^^^^^^^^^^^^^^^^^^^^^^^^^^^^^^^^^^^^^^^^^^^^^^^^^^^^^^^^^^^^^^
5. The manuscript provides mean performance metrics, but what is the variability (e.g., standard deviation, confidence intervals) across different cross-validation folds? Reporting this would offer a more complete picture of model robustness.

**Reply**

**In addition, we have conducted a more comprehensive study by selecting the number of principal components (PCs) in the range of 4 to 6, for the classification analysis. Table 2 presents the results of the 10 performance metrics for the training set, evaluated using five-fold cross-validation on the balanced training set, along with their standard deviations, providing a comprehensive view of the model's robustness.**

**The last row of Table 2 presents the comparative scores obtained by evaluating the 10 metrics, where higher values indicate better performance. We observed that NQE+QSVC outperforms NQE+SVC when using 5 and 6 PCs ‘test’ set results (comparative score is 0 vs. 10). However, NQE+SVC achieves superior performance with 4 PCs. These results suggest that NQE has the potential to improve the performance of QSVC classifiers. Additionally, all standard deviation values are relatively small in comparison to the mean values, except in five cases (NQE+SVC, 4PCs – SPC, FPR, FNR, and MCC; and NQE+SVC, 5PCs – SPC), indicating that the results for the 10 performance metrics are generally robust.**

**We also observed a significant difference between the performance metric values for the training and test sets, particularly for PPV, F1, FDR, FNR, and MCC. However, this difference is less pronounced when data balancing techniques are applied, compared to the scenario without data balancing, where the discrepancy between training and test set results is much more pronounced (Table 2). This suggests that the issue of data imbalance remains an inherently challenging problem.**

Table 2. Classification performance results using 5-fold cross-validation for the training datasets after applying the data imbalance tool, SMOTE. The values without the '±' signs represent the results for the ‘test’ set.

|  | 4 PCs | | 5 PCs | | 6 PCs | |
| --- | --- | --- | --- | --- | --- | --- |
| Metrics | NQE+SVC | NQE+QSVC | NQE+SVC | NQE+QSVC | NQE+SVC | NQE+QSVC |
| Sensitivity/Recall, TPR | 0.674$\pm0.240,$  0.5 | 0.707$\pm0.058,$  0.46 | 0.856$\pm0.144,$  0.36 | 0.81$\pm0.033,$  0.46 | 0.839$\pm0.21,$  0.4 | 0.819$\pm0.061,$  0.5 |
| Specificity, SPC | 0.543$\pm0.348,$  0.67 | 0.828$\pm0.064$,  0.64 | 0.29$\pm0.193,$  0.70 | 0.359$\pm0.094,$  0.71 | 0.678$\pm0.229,$  0.66 | 0.765$\pm0.054,$  0.69 |
| Precision, PPV | 0.638$\pm0.13,$  0.25 | 0.807$\pm0.054,$  0.22 | 0.552$\pm0.038,$  0.21 | 0.561$\pm0.034,$  0.26 | 0.743$\pm0.099,$  0.20 | 0.779$\pm0.042,$  0.26 |
| F1 Score | 0.612$\pm0.15,$  0.33 | 0.752$\pm0.044,$  0.30 | 0.66$\pm0.089,$  0.27 | 0.585$\pm0.043,$  0.34 | 0.758$\pm0.188,$  0.27 | 0.797$\pm0.042,$  0.34 |
| Accuracy | 0.608$\pm0.089,$  0.64 | 0.767$\pm0.042,$  0.61 | 0.573$\pm0.049,$  0.64 | 0.662$\pm0.024,$  0.66 | 0.758$\pm0.11,$  0.61 | 0.79$\pm0.041,$  0.66 |
| Negative Predictive Value (NPV) | 0.649$\pm0.123,$  0.86 | 0.739$\pm0.040,$  0.85 | 0.747$\pm0.155,$  0.84 | 0.648$\pm0.063,$  0.86 | 0.84$\pm0.099,$  0.84 | 0.812$\pm0.052,$  0.86 |
| False Positive Rate (FPR)* | 0.457$\pm0.348,$  0.33 | 0.172$\pm0.064,$  0.35 | 0.709$\pm0.155,$  0.29 | 0.64$\pm0.094,$  0.28 | 0.322$\pm0.23,$  0.33 | 0.235$\pm0.054,$  0.30 |
| False Discovery Rate (FDR) * | 0.36$\pm0.13,$  0.75 | 0.193$\pm0.054,$  0.77 | 0.448$\pm0.038,$  0.78 | 0.439$\pm0.034,$  0.74 | 0.256$\pm0.099,$  0.79 | 0.22$\pm0.042,$  0.74 |
| False Negative Rate (FNR)* | 0.33$\pm0.13,$  0.5 | 0.293$\pm0.058,$  0.53 | 0.144$\pm0.144,$  0.63 | 0.189$\pm0.033,$  0.53 | 0.16$\pm0.211,$  0.6 | 0.18$\pm0.061,$  0.5 |
| Matthews Correlation Coefficient (MCC) | 0.278$\pm0.159,$  0.14 | 0.541$\pm0.082,$  0.09 | 0.201$\pm0.077,$  0.06 | 0.188$\pm0.088,$  0.15 | 0.597$\pm0.112,$  0.05 | 0.587$\pm0.082,$  0.16 |
| Comparative scores | 10 | 0 | 0 | 10 | 0 | 10 |

* A lower value implies better performance

6. The manuscript notes that the M0 samples (non-metastasis) are 5.44 times larger than M1 samples (metastasis). Have you considered performing analyses without data balancing to compare results with and without SMOTE to assess the real impact of data imbalance? What measures did you take to ensure that SMOTE did not introduce bias?

**Reply**

**Without applying SMOTE to address the data imbalance, classification performance using 10, 11, and 12 principal components (PCs) is problematic. A key observation is that specificity is perfect (1.0) for both 10 and 12 PCs, meaning the classifier correctly identifies all true negatives (TN, non-metastatic cases, M0). However, recall is very low, dropping as low as 0.03, indicating that the classifier is only able to identify a small fraction of metastatic cases (M1). Given these results, we decided to apply the SMOTE technique to address the data imbalance issue in our current work.**

**The following results are not included in the revised manuscript; they are listed here to address the comment.**

Table. Classification performance results for the test dataset with optimal hyperparameters, C and gamma, *without* applying the data imbalance tool, SMOTE.

| Metrics | 10 PCs | 11 PCs | 12 PCs |
| --- | --- | --- | --- |
| Sensitivity/Recall, TPR | 0.03 | 0.06 | 0.03 |
| Specificity, SPC | 1.00 | 0.99 | 1.00 |
| Accuracy | 0.83 | 0.83 | 0.83 |
| F1 Score | 0.06 | 0.12 | 0.06 |

**We also have studied the results of using NQE without addressing data imbalance (mRNA expression data using 4 PCs). The results are unstable; for example, there is a significant variation in the F1 Score, ranging from 0 to 0.062, and the MCC can even be negative.**

**The following results are not included in the revised manuscript; they are listed here to address the comment.**

Table. Classification performance results for the test dataset (mRNA using 4 PCs) *with and* *without* applying the data imbalance tool, SMOTE.

|  | *With SMOTE* | | *Without SMOTE* | |
| --- | --- | --- | --- | --- |
| Metrics | NQE+SVC | NQE+QSVC | NQE+SVC | NQE+QSVC |
| Sensitivity/Recall, TPR | 0.5 | 0.5 | 0.0 | 0.03 |
| Specificity, SPC | 0.65 | 0.69 | 0.99 | 0.99 |
| Precision, PPV | 0.24 | 0.26 | 0.00 | 0.5 |
| F1 Score | 0.32 | 0.34 | 0.00 | 0.062 |
| Accuracy | 0.62 | 0.66 | 0.82 | 0.82 |
| Negative Predictive Value | 0.85 | 0.86 | 0.82 | 0.83 |
| False Positive Rate * | 0.35 | 0.30 | 0.007 | 0.007 |
| False Discovery Rate * | 0.76 | 0.74 | 1.0 | 0.5 |
| False Negative Rate * | 0.5 | 0.5 | 1.0 | 0.96 |
| Matthews Correlation Coefficient | 0.12 | 0.16 | -0.036 | 0.092 |
| Comparative Points | 0 | 9 and 1-tier | 0 | 10 |

^^^^^^^^^^^^^^^^^^^^^^^^^^^^^^^^^^^^^^^^^^^^^^^^^^^^^^^^^^^^^^^^^^^^^^^^^^^

7. Why did you select principal components ranging from 9 to 12 for the gene expression study? Did you experiment with other numbers of principal components, and how did those results compare? Could you explain more about the rationale behind this choice?

**Reply**

Here is the updated paragraph in the revised manuscript.

**The cumulative contribution of the first four, five, six, nine and 12 largest eigenvalues amounts to 52.4%, 56.4%, 59.2%, 69.1% and 74.8%, respectively, of the total sum of 48 eigenvalues from the covariance matrix. This means that more than 50% of the variance is explained by these eigenvalues. The values indicate the proportion of total variance explained by the first 4 to 12 principal components. Higher values indicate that the corresponding principal components capture more of the variance in the data, while lower values suggest less contribution to the overall variance.**

^^^^^^^^^^^^^^^^^^^^^^^^^^^^^^^^^^^^^^^^^^^^^^^^^^^^^^^^^^^^^^^^^^^^^^^^^^^
8. The study focuses on two specific datasets (gene expression and miRNA profiles). Have you tested the NQE+QSVC model on other biomedical datasets or non-biomedical data to assess its generalizability?

**Reply**

**The following results are not included in the revised manuscript; they are listed here to address the comment.**

The application of NQE to enhance machine learning classification was initially explored with non-biomedical data, specifically the MNIST and FashionMNIST datasets. The results indicate that combining NQE with QSVC improves classification performance. In contrast, using QSVC alone yields inferior results compared to SVC. The detailed results are presented in the table below.

**Table.** Classification accuracy of the MNIST and FashionMNIST datasets using three classification methods: (i) QSVC, (ii) SVC, and (iii) NQE + QSVC.

| classifier | Number of features | MNIST | Fashion MNIST |
| --- | --- | --- | --- |
| QSVC | 2 | 59.60$\pm6$.25% | 47.20$\pm6.01\%$ |
|  | 4 | 73.20$\pm2.71\%$ | 70.40±11.13% |
|  | 8 | 63.20$\pm5.31\%$ | 72.80$\pm3.49\%$ |
| SVC | 2 | 82.80$\pm4.83\%$ | 69.20$\pm0.98\%$ |
|  | 4 | 97.60$\pm3.88\%$ | 92.00$\pm2.53\%$ |
|  | 8 | 98.80$\pm0.98\%$ | 94.80$\pm2.04\%$ |
| NQE+QSVC | 2 | 83.20$\pm4.31\%$ | 69.20±0.98% |
|  | 4 | 97.60$\pm3.88\%$ | 88.80$\pm4.83\%$ |
|  | 8 | 98.80$\pm1.60\%$ | 93.20$\pm5.60\%$ |

The above work earned the ‘Enterprise Special Prize’ at the Qiskit Hackathon Taiwan 2023. Details can be found at <https://quantum.ntu.edu.tw/?p=8948>, under the paragraph beginning with 'The ninth team won an Enterprise Special Prize with the topic “Classical Machine Learning for Quantum Computing.”' The manuscript’s first author, Miss Aninda Astuti, was a member of the award-winning team.

Motivated by the results of applying NQE to non-biomedical datasets (not detailed in this manuscript), we extended the approach to molecular medicine data. This adaptation demonstrated that NQE combined with QSVC effectively enhances classification performance, highlighting its broad applicability across diverse domains.

^^^^^^^^^^^^^^^^^^^^^^^^^^^^^^^^^^^^^^^^^^^^^^^^^^^^^^^^^^^^^^^^^^^^^^^^^^^

9. How does the use of SMOTE (Synthetic Minority Oversampling Technique) affect the results, especially in terms of potential overfitting? Have you considered alternative data balancing techniques (e.g., undersampling or cost-sensitive learning), and how would they compare to SMOTE in your study?

**Reply**

**As demonstrated above, the differences are less pronounced when data balancing techniques are applied. In contrast, without data balancing, the discrepancies among various metrics, such as TPR, SPC, F1, and MCC, are much more pronounced. This suggests that without data balancing, the model encounters overfitting issues. The issues associated with using undersampling or cost-sensitive learning for data balancing remain to be investigated.**

^^^^^^^^^^^^^^^^^^^^^^^^^^^^^^^^^^^^^^^^^^^^^^^^^^^^^^^^^^^^^^^^^^^^^^^^^^^

For Future Work:
1. Do you have plans to implement the QSVC model on real quantum hardware (e.g., IBM Q or other NISQ devices)? If so, how do you plan to address potential challenges like noise and qubit limitations?

**Reply**

**Another limitation is that simulating quantum circuits on classical computers is constrained by scalability issues. Even when encoding features with only a few quantum qubits, the process remains highly time-consuming. A promising solution involves using tensor networks to represent quantum circuits. To address this, we plan to accelerate the QSVC implementation with NVIDIA's cuQuantum package [75].**

^^^^^^^^^^^^^^^^^^^^^^^^^^^^^^^^^^^^^^^^^^^^^^^^^^^^^^^^^^^^^^^^^^^^^^^^^^^

2. Given that your study is focused on improving metastasis classification, how do you envision your results being used in clinical settings? Are there particular areas in precision medicine where this hybrid quantum approach could have an immediate impact?

**Reply**

**Over several decades, clear cell renal cell carcinoma (ccRCC) has been the predominant subtype of renal cancer, accounting for 80-90% of renal cases and remaining a highly lethal urological malignancy. Metastasis, the final stage of cancer, remains a challenging area of study, with the precise mechanisms behind cancer cell dissemination and establishment at distant sites still largely unknown. In this context, both gene and microRNA (miRNA) expression profiles are used to classify tumor metastasis, offering a more accurate and comprehensive patient classification. This approach aligns with the principles of precision medicine, tailoring treatments based on molecular profiles to improve outcomes. By integrating both mRNA and miRNA data, this work enhances our understanding of cancer metastasis and supports the development of more personalized therapeutic strategies for ccRCC and other cancers.**

^^^^^^^^^^^^^^^^^^^^^^^^^^^^^^^^^^^^^^^^^^^^^^^^^^^^^^^^^^^^^^^^^^^^^^^^^^^

Reviewer #2: The research explores the application of QML in identifying gene expression biomarkers for metastasis in ccRCC, a highly lethal form of renal cancer. The study focuses on improving execution time and accuracy in binary classification using the Quantum Support Vector Classifier compared to the classical SVC. Inspired by the Neural Quantum Embedding (NQE) method, they propose a two-stage approach to evaluate whether combining NQE with QSVC outperforms NQE with SVC across various biomedical datasets.
1. The organization of the paper needs to be improved, especially the Methods and Results sections.

**Reply**

**In the revised manuscript, the 'Introduction' section now includes more detailed descriptions to explain the underlying principles of quantum computing.**

**The 'Materials and Methods' and 'Results' sections have been updated with more detailed explanations of Figures 1 to 4. For Figure 1, we have made a few changes: (1) corrected a typo (test set: 1/3), (2) change ‘Dimension Reduction (PCA 9.10,11,12) to Dimension Reduction (PCA 4 – 6, 9 - 12). (3) The figure caption has revised.**

**For Figure 2, it has revised with the addition of the conjugate circuit layer. Also, we have provided a more detailed description of the quantum embedding circuit in the revised manuscript. This additional information has been included after the following paragraph: ‘The Neural Quantum Embedding (NQE) approach … capable of separating the  classes of data into orthogonal subspaces.’**

**For Figure 3, we included an additional paragraph to introduce the concept of NQE. For Figure 4, we added an additional paragraph to explain the meaning of the loss function.**

^^^^^^^^^^^^^^^^^^^^^^^^^^^^^^^^^^^^^^^^^^^^^^^^^^^^^^^^^^^^^^^^^^^^^^^^^^^
2. Although the article uses quantum computing and an interesting dataset, the research method lacks novelty in both the data embedding part and the quantum support vector machine. I am more looking forward to seeing improvements and enhancements to these methods.

**Reply**

**The application of NQE to enhance machine learning classification was initially explored with non-biomedical data, specifically the MNIST dataset. The results indicate that combining NQE with a Quantum Convolutional Neural Network (QCNN) improves classification performance. However, there is no comparison with NQE+CNN to determine if it yields inferior results compared to the NQE+QCNN architecture.**

**We explored the application of NQE to enhance machine learning classification in four ways: (i) using two types of biomedical data, (ii) addressing data imbalance issues, (iii) comparing NQE+QSVC with NQE+SVC, and (iv) comparing NQE+QNN with NQE+NN. This allows us to determine whether machine learning classification algorithms yield inferior results without the use of NQE compared to when NQE is utilized. Our results indicate that the NQE approach can be extended to molecular medicine data, demonstrating its potential as a general method to enhance classification performance. This study does not aim to improve the data embedding process or the quantum machine learning algorithms.**

^^^^^^^^^^^^^^^^^^^^^^^^^^^^^^^^^^^^^^^^^^^^^^^^^^^^^^^^^^^^^^^^^^^^^^^^^^^
3. Figure 1 and figure 2 need to be improved, they are not very clear at the moment.

**Reply**

**In the revised manuscript, for Figure 1, we have made a few changes: (1) corrected a typo (test set: 1/3), (2) change ‘Dimension Reduction (PCA 9.10,11,12) to Dimension Reduction (PCA 4 – 6, 9 - 12). (3) The figure caption has revised. For Figure 2, it has revised with the addition of the conjugate circuit layer. Also, we have provided a more detailed description of the quantum embedding circuit in the revised manuscript. This additional information has been included after the following paragraph: ‘The Neural Quantum Embedding (NQE) approach … capable of separating the  classes of data into orthogonal subspaces.’**

^^^^^^^^^^^^^^^^^^^^^^^^^^^^^^^^^^^^^^^^^^^^^^^^^^^^^^^^^^^^^^^^^^^^^^^^^^^
4. There is some lack of mathematical description of quantum support vector machines.

**Reply**

**We provided more mathematical descritpion of QSVC in the revised manuscript.**

**The QSVC in the IBM Qiskit package is used to handle the two classification problems by leveraging the computational advantages of quantum computers. The QSVC inherits methods like ‘fit’ and ‘predict’ from Scikit-learn. The QSVC uses a quantum kernel, named ‘FidelityQuantumKernel’, for computing the kernel matrix, which can capture complex patterns in the data by leveraging quantum computation.**

**The quantum kernel in QSVC is determined by calculating the inner product of the quantum states that encode the data, which is given by**

$\mathbf{K}\left( \mathbf{x,z} \right)\mathbf{=}\left| {}^{\bigotimes\mathbf{n}}\mathbf{<}\mathbf{0|}\mathbf{U}^{\boldsymbol{\dagger}}\mathbf{(z)U(x)} \right|\mathbf{0}\mathbf{>}^{\bigotimes\mathbf{n}}\mathbf{|}^{\mathbf{2}}$**. (7)**

**where** $\mathbf{U(x)}$ **represents the unitary operation derived from the application of a layer of Hadamard gates followed by the quantum feature map using NQE method,** $\mathbf{V}\left( \boldsymbol{\varphi}\left( \mathbf{x} \right) \right)$**, here** $\mathbf{x}$ **and** $\mathbf{z}$ **are the input data. The kernel is computed by selecting** $\mathbf{U}\left( \mathbf{x} \right)$ **and iterating through the other state** $\mathbf{U}^{\boldsymbol{\dagger}}\mathbf{(z)}$**. When** $\mathbf{U}\left( \mathbf{x} \right)$ **is close to** $\mathbf{U}\left( \mathbf{z} \right)$**, the circuit exhibits a large transition amplitude between states** $\mathbf{U}^{\boldsymbol{\dagger}}\left( \mathbf{z} \right)\mathbf{U}\left( \mathbf{x} \right)\mathbf{|0}\mathbf{>}^{\bigotimes\mathbf{n}}$**and** ${}^{\bigotimes\mathbf{n}}\mathbf{<}\mathbf{0|}$**, where** $\mathbf{|0>}$ **is the computational z-basis. When the measurement is repeated, the frequency of the all** $\mathbf{|0}\mathbf{>}^{\bigotimes\mathbf{n}}$ **state approximates the quantum kernel** $\mathbf{K}\left( \mathbf{x,z} \right)$**. Once this kernel is trained, it can be used to compute the kernel function between the training and test set, facilitating classification.**

**We note that the potential advantage of using quantum kernels lies in their ability to leverage computational features that are difficult to simulate efficiently on classical systems. However, this advantage alone is a necessary condition but it is not sufficient [74].**

^^^^^^^^^^^^^^^^^^^^^^^^^^^^^^^^^^^^^^^^^^^^^^^^^^^^^^^^^^^^^^^^^^^^^^^^^^^
5. The loss function in figure 4 is quite confusing and I don’t know what it reflects.

**Reply**

**Figure 4 illustrates the plots of the training loss (PyTorch mean squared error package, *MSEloss*) as a function of the number of iterations for 9 to 12 principal components (PCs) during the training phase of the NQE method with a classical NN. This process resulted in an updated weight for the function 𝑔(𝒙, 𝒘).**

6. PLOS authors have the option to publish the peer review history of their article ([what does this mean?](https://track.editorialmanager.com/CL0/https:%2F%2Fjournals.plos.org%2Fplosone%2Fs%2Feditorial-and-peer-review-process%23loc-peer-review-history/1/010f0192fe46903d-c7004a41-df1f-4cc0-997b-cb76a377d91f-000000/FSs9xTfRzC3ls8_PRYlAdXHSjxL5MRZCVgub_lzEtos=183)). If published, this will include your full peer review and any attached files.
If you choose “no”, your identity will remain anonymous but your review may still be made public.

**Do you want your identity to be public for this peer review?** For information about this choice, including consent withdrawal, please see our [Privacy Policy](https://track.editorialmanager.com/CL0/https:%2F%2Fwww.plos.org%2Fprivacy-policy/1/010f0192fe46903d-c7004a41-df1f-4cc0-997b-cb76a377d91f-000000/UUJQUIThoLcz7b8mnPdoljf0UwghuoiaLcqyj3BF9V4=183).

Reviewer #1: No

Reviewer #2: No

[NOTE: If reviewer comments were submitted as an attachment file, they will be attached to this email and accessible via the submission site. Please log into your account, locate the manuscript record, and check for the action link "View Attachments". If this link does not appear, there are no attachment files.]
While revising your submission, please upload your figure files to the Preflight Analysis and Conversion Engine (PACE) digital diagnostic tool, [https://pacev2.apexcovantage.com/](https://track.editorialmanager.com/CL0/https:%2F%2Fpacev2.apexcovantage.com%2F/1/010f0192fe46903d-c7004a41-df1f-4cc0-997b-cb76a377d91f-000000/LeENxlpK7gKsHZLl2ChElBmJOw4Sp12nrTchAQAlylk=183). PACE helps ensure that figures meet PLOS requirements. To use PACE, you must first register as a user. Registration is free. Then, login and navigate to the UPLOAD tab, where you will find detailed instructions on how to use the tool. If you encounter any issues or have any questions when using PACE, please email PLOS at [figures@plos.org](mailto:figures@plos.org). Please note that Supporting Information files do not need this step.
