# Supplementary material for: Use of hybrid quantum-classical algorithms for enhancing biomarker classification
Source: PLoS One. 2025 Jul 17;20(7):e0327928. doi: 10.1371/journal.pone.0327928 (PMC12270134; doi:10.1371/journal.pone.0327928)
Supplement: Attachment — Submitted filename: reply-qsvc-qnn-R2-v3.docx [file pone.0327928.s018.docx]

**Responses to Reviewers' comments**

Reviewer's Responses to Questions

**Comments to the Author**

1. If the authors have adequately addressed your comments raised in a previous round of review and you feel that this manuscript is now acceptable for publication, you may indicate that here to bypass the “Comments to the Author” section, enter your conflict of interest statement in the “Confidential to Editor” section, and submit your "Accept" recommendation.

Reviewer #1: All comments have been addressed

Reviewer #3: (No Response)

**Reply**

**We thank the reviewers for their helpful suggestions, which have contributed to improving our work.**

2. Is the manuscript technically sound, and do the data support the conclusions?

The manuscript must describe a technically sound piece of scientific research with data that supports the conclusions. Experiments must have been conducted rigorously, with appropriate controls, replication, and sample sizes. The conclusions must be drawn appropriately based on the data presented.

Reviewer #1: Yes

Reviewer #3: Partly

**Reply**

**We thank the reviewers for their helpful suggestions, which have contributed to improving our work. In the revised manuscript, we expand upon the previous analysis by incorporating NQE with QSVC/QNN to analysis a third dataset.**

3. Has the statistical analysis been performed appropriately and rigorously?

Reviewer #1: (No Response)

Reviewer #3: N/A

**Reply**

**We thank the reviewers for their helpful suggestions, which have contributed to improving our work.**

4. Have the authors made all data underlying the findings in their manuscript fully available?

The [PLOS Data policy](http://track.editorialmanager.com/CL0/http:%2F%2Fwww.plosone.org%2Fstatic%2Fpolicies.action%23sharing/1/010f01963f02475f-4bb361a0-6b26-466f-8722-c14cfc506bc7-000000/VvcDvRgU3gruG9aoDkkOIBwsBaF0ZjfWofLgBK9BhRs=206) requires authors to make all data underlying the findings described in their manuscript fully available without restriction, with rare exception (please refer to the Data Availability Statement in the manuscript PDF file). The data should be provided as part of the manuscript or its supporting information, or deposited to a public repository. For example, in addition to summary statistics, the data points behind means, medians and variance measures should be available. If there are restrictions on publicly sharing data—e.g. participant privacy or use of data from a third party—those must be specified.

Reviewer #1: Yes

Reviewer #3: No

**Reply**

**We thank the reviewers for their helpful suggestions, which have contributed to improving our work. We uploaded the three input datasets and the codes for NQE+QSVC and NQE+QNN to GitHub,** [**https://github.com/anindaastuti/NQE-QSVC-QNN**](https://github.com/anindaastuti/NQE-QSVC-QNN)**.**

5. Is the manuscript presented in an intelligible fashion and written in standard English?

PLOS ONE does not copyedit accepted manuscripts, so the language in submitted articles must be clear, correct, and unambiguous. Any typographical or grammatical errors should be corrected at revision, so please note any specific errors here.

Reviewer #1: Yes

Reviewer #3: Yes

**Reply**

**We thank the reviewers for their helpful suggestions, which have contributed to improving our work.**

6. Review Comments to the Author

Please use the space provided to explain your answers to the questions above. You may also include additional comments for the author, including concerns about dual publication, research ethics, or publication ethics. (Please upload your review as an attachment if it exceeds 20,000 characters)

Reviewer #1: Dear Authors,
Thank you for addressing my previous comments and significantly improving the manuscript. However, I have a few minor suggestions to further enhance the clarity and reproducibility of your work.
- Please provide a direct link about the dataset used in this study to facilitate reproducibility.
- Consider sharing the implementation code (e.g., via GitHub or a public repository) to support further validation and adoption of your approach.

**Reply**

**We uploaded the three input datasets and the codes for NQE+QSVC and NQE+QNN to GitHub,** [**https://github.com/anindaastuti/NQE-QSVC-QNN**](https://github.com/anindaastuti/NQE-QSVC-QNN)**.**

^^^^^^^^^^^^^^^^^^^^^^^^^^^^^^^^^^^^^^^^^^^^^^^^^^^^^^^^^^^^^^^^^^^^^^^

Reviewer #3: 1. The revised manuscript has addressed some reviewer comments; however, significant issues remain.

First, the explanation of the training loss (as presented in Figure 4) is still too terse. The manuscript merely states that the loss decreases over iterations and “results in an updated weight for the function ( , )” without providing an intuitive explanation of what this loss represents regarding convergence or overall model performance. Please expand this discussion, clarifying how the loss behavior reflects improvements in training and its implications for the robustness of the quantum embedding.

**Reply**

**We appreciate the reviewer’s valuable suggestions, which have helped enhance our work. In the revised manuscript, we have included a more detailed explanation of the loss function. Please see the three paragraphs above Figure 5.**

2. Although the authors have added details regarding the quantum embedding circuit, the description remains highly verbose. It is recommended that the authors provide a more accessible, **top-level overview of the embedding process**. This should include a schematic or algorithmic flow diagram that distinguishes clearly between the novel contributions (e.g., the integration of NQE with QSVC/QNN) and the components based on existing methods, along with a discussion of the design decisions made.

**Reply**

**We thank the reviewer’s valuable suggestions, which have helped improve our work. In the revised manuscript, we have included a more detailed explanation of the the embedding process. Please see lines 215 to 264 for further details.**

**Figure 2 (newly added) presents a flow diagram illustrating the integration of NQE with QSVC and QNN. A detailed discussion of the underlying design decisions can be found in lines 268-283 for NQE+QSVC, and lines 343-351 for NQE+QNN.**

3. The manuscript still suffers from language and organizational issues. Several sections are laden with technical jargon and complex phrasing, which hinders clarity. A thorough language and structural revision is necessary to improve readability and ensure consistency throughout the text.

**Reply**

**We thank the reviewer’s valuable suggestions, which have helped improve our work. We have thoroughly revised the manuscript, using simple English and ensuring that all sentences are no longer than two lines.**

**Please refer to the highlighted text (manuscript with tracked changes) in the following sections, which indicate the revisions: Introduction, Materials and Methods, Results, Discussion, and Conclusion.**

4. The presentation of the experimental results, especially in Tables 1–3 and Figures 4–8, would benefit from a more detailed discussion. Each figure and table should be accompanied by a list of key observation points that help the reader interpret the significance of the performance metrics. This would also aid in understanding the practical impact of the proposed method.

**Reply**

**We thank the reviewer’s valuable suggestions, which have helped improve our work. We have thoroughly revised the presentation of the experimental results.**

**Please refer to the highlighted text (manuscript with tracked changes) in the following items, which indicate the revisions: Table 1, Table 2, Table 3, Conclusion, and the updated figure numbering — Figure 4 is now Figure 5, Figure 5 is now Figure 6, Figure 6 is now Figure 7, Figure 7 is now Figure 8, and Figure 8 is now Figure 9.**

5. The description of the experimental setup and workflow—including the use of data preprocessing techniques (such as SMOTE and PCA), hyperparameter tuning, and the simulation of quantum circuits—remains insufficient. More details should be provided to allow reproducibility. It is strongly encouraged that the authors share their source code via an online repository.

**Reply**

**We uploaded the three input datasets and the codes for NQE+QSVC and NQE+QNN to GitHub,** [**https://github.com/anindaastuti/NQE-QSVC-QNN**](https://github.com/anindaastuti/NQE-QSVC-QNN)**.**

6. While the manuscript compares the performance of NQE+QSVC versus NQE+SVC (and similarly NQE+QNN versus NQE+NN), it would be valuable to include a discussion of how these hybrid approaches compare with more conventional classical methods beyond the SVC and NN models presented. Please discuss the limitations of the related work and explain in detail how the proposed framework overcomes these issues.

**Reply**

**We appreciate the reviewer's insightful comments regarding the comparison of our hybrid approaches with more conventional classical methods beyond SVC and NN, and ask for a discussion on the limitations of related work. The manuscript has been thoroughly revised; see Table 4 and the two paragraphs above for details**

7. The current experimental evaluation is limited to two datasets (gene expression and miRNA expression profiles for ccRCC metastasis). To demonstrate the generalizability of the proposed method, the authors should consider applying their framework to a broader range of more challenging benchmarks.

**Reply**

**In the revised manuscript, we evaluate how well our method works, we applied it to a third dataset. Please refer to the two paragraphs above the ‘Discussion’ section, and review Supplementary Files 4 and 5, as well as Supplementary Figures 5 to 10, for details.**

8. Finally, please discuss the potential impact of this work at a larger scale and outline possible future research directions. Addressing how this hybrid quantum-classical approach could be applied to other domains or scaled up for more complex applications would significantly strengthen the manuscript.

**Reply**

**We thank the reviewer’s valuable suggestions, which have helped improve our work. To address this, we plan to accelerate the QSVC implementation with NVIDIA's cuQuantum package [77]. Our goal is to enhance computational efficiency by integrating Tensor Network (TN) techniques into the QSVC implementation. This future work could demonstrate the potential of combining QML with tensor-based optimization for efficient and accurate analysis of high-dimensional biomedical data. Please refer to the paragraph above the ‘Conclusion’ section**

**The following paragraph is not included in the revised manuscript.**

**We have recently integrated Tensor Network (TN) techniques into the QSVC implementation and submitted the resulting work to an international conference, where it is currently under peer review. Our prelminary results show that, using 12 principal components (PCs), the F1 score of the NQE+TN+QSVC model improves by a factor of 2.02 compared to NQE+QSVC and by 2.15 compared to NQE+SVC. Furthermore, the integration of TN reduces the QSVC training time by up to 21.82-fold when using 12 PCs.**
